# COVID-19-Related Transformations and Post-Pandemic Recovery for Informal Food Vendors in Secondary Cities: A Case of Kisumu City, Kenya

**DOI:** 10.3390/ijerph23050575

**Published:** 2026-04-29

**Authors:** Silvia Achieng Odhiambo, Patrick Mbullo Owuor, Doreen Obondo, Janet Anyango Onyango, Elizabeth Onyango

**Affiliations:** 1 School of Public Health, University of Alberta, Edmonton, AB T6G 1C9, Canada; saodhiam@ualberta.ca (S.A.O.); jonyango@ualberta.ca (J.A.O.); 2 Pamoja Community-Based Organization, Kisumu 2311-40100, Kenya; hp4113@wayne.edu (P.M.O.); d.obondo@gmail.com (D.O.); 3 Department of Anthropology, Wayne State University, Detroit, MI 48202, USA

**Keywords:** COVID-19 pandemic, informal female food vendors, food insecurity, gender-based violence, resilience strategies, post-pandemic recovery, economic vulnerability

## Abstract

**Highlights:**

**Public health relevance—How does this work relate to a public health issue?**

- Female informal food vendors play a key role in supporting urban household food security and livelihood needs
- The pandemic induced changes in the market, household system and in gender roles disproportionately impacted informal female food vendors in secondary cities in the global South

**Public health significance—Why is this work of significance to public health?**

- This study provides evidence on the structural and gender inequities within the informal sector, particularly in times of emergency
- The female informal food vendors adopted various coping strategies including individual and collective agencies to ensure resilience to the pandemic impact

**Public health implications—What are the key implications or messages for practitioners, policy makers and/or researchers in public health?**

- Community-based organizations can effectively address local needs and strengthen future crisis response and recovery
- During the pandemic while grassroots collaboration proved effective, integrating these efforts with institutional support can enhance sustainability and equity

**Abstract:**

While substantial literature exists on COVID-19’s effects on businesses, long-term recovery strategies and support systems for informal female-owned enterprises in secondary cities are underexplored. The study sought to qualitatively examine the gendered impacts of COVID-19 on informal food businesses owned by women in Kenya. Qualitative interviews with 80 participants, including key informant interviews, in-depth interviews, and focus group discussions in Kisumu, Kenya, were conducted. The study found that the pandemic significantly disrupted the livelihoods of female vendors, leading to changes in the market and household organization, including gender specific transformations. The women adopted some individual and collective strategies as part of the post-pandemic recovery strategies to enhance their resilience in business. The study findings shed light on the vulnerabilities of informal food businesses in secondary cities to emergencies and the need for targeted policies to support informal economies during crises.

## 1. Introduction

Food security remains a global concern, particularly in urban centers in the global South [1]. In 2021, an estimated 29.3% of the global population—2.3 billion people—were moderately or severely food insecure, with women disproportionately affected at 31.9% compared to 27.6% of men [1]. In sub-Saharan Africa, 66.2% of the population had moderate to severe food insecurity in 2021, with women and children being the most affected by undernutrition [2]. Undernutrition in Africa rose from 17.6% to about 20% in 2019, and with the current global emergencies, including lingering effects of the COVID-19 pandemic, the geopolitics, climate change, and global wars across the world, the prevalence is predicted to be on the rise in most African countries [2,3,4]. With the prevailing emergencies, gender disparity in food security is particularly dire in cities in sub-Saharan Africa, and informal vendors are playing a significant role in addressing some of these challenges [2].

Informal food vending constitutes a vital segment of urban food security in resource-poor regions, supporting the livelihoods of millions while providing essential food access to low-income urban populations [5]. The sector provides jobs, especially for women and marginalized groups such as youth, who struggle to access formal employment [6]. Informal food vendors provide diverse, culturally appropriate, and affordable food options in urban settings where supermarkets and formal retail are often inaccessible or unaffordable to most households [7]. By sourcing directly from local farmers and suppliers, and selling in smaller quantities, these vendors minimize food waste and enhance food system flexibility [8]. Women food vendors, in particular, play crucial roles as income earners for their households, using their earnings to support children’s education, healthcare, and basic needs [9,10]. Despite these contributions, the sector faces persistent social and structural challenges, including limited financial support, lack of policy recognition, infrastructural constraints, and exposure to external shocks—such as the COVID-19 pandemic [11,12].

The COVID-19 pandemic’s containment measures, including lockdowns, travel restrictions, and market closures, disrupted urban food systems globally, with particularly severe consequences for informal female food vendors [13,14]. Female informal food vendors faced added pressures as they dealt with business disruptions while also taking on greater caregiving roles at home, including childcare during school closures, caring for sick relatives, and more household work [15,16]. This double burden of unpaid work and household responsibilities, long experienced by women in informal economies, became much heavier during the pandemic, leaving many unable to keep their businesses running and/or meet the needs of their families [17]. A study in Nairobi found that 86% of city’s residents experienced income loss due to COVID-19 lockdowns, and 74% reported eating less or skipping meals because of inadequate income [18]. For female vendors dependent on daily earnings, these disruptions translated into immediate household food insecurity, economic crisis, and added responsibilities [19].

In Kisumu, Kenya’s third-largest city, informal food businesses play a crucial role in providing employment for women within the informal sector, with over 75% of the food consumed in the city obtained from informal female traders [20]. Despite the growth of supermarkets and shopping malls, street food vending remains essential to the city and for households in Kisumu’s informal settlements who rely on these businesses for their daily necessities [20,21,22,23]. This sector provides affordable and accessible food options for urban households, addressing the population’s dietary needs [8,24,25,26,27]. Despite their key role in food security and livelihoods, informal food vendors in Kisumu receive little support from the government [24,25].

Before the pandemic, Kisumu County regulations focused primarily on revenue collection through daily market fees and levies rather than vendor support, infrastructure provision, or integration into formal food system planning [21,28,29]. Vendors had no access to formal credit, market infrastructure was inadequate, with many sites lacking water, sanitation, and covered vending areas, and the sector received little attention in county development planning [20,30]. This regulatory approach viewed informal vendors as problems to control rather than as contributors to urban food security and livelihoods. Because they were not formally recognized, vendors lacked a voice in policymaking, access to government credit or business support, and social protection during pandemic crises [24].

In Kisumu city, the informal food sector operates within overlapping contexts of gender-based violence (GBV) and patriarchal norms that deeply shape women’s experiences during crises [21]. Over 60% of the informal food businesses in Kisumu are female-led, most of whom identify as either Luo- or Luhya-speaking communities, where females (women and girls) are less valued, and gender roles are societally defined. Studies in the city show high rates of intimate partner violence among women [31], particularly those in the informal sector and more so, during times of economic stress when poverty increases, and men often control women’s earnings, creating tension and conflict [32]. These patterns reflect dominant masculinity norms that link the identity of men to being family providers, especially within communities in neighbouring Kisumu City. When economic crises weaken their ability to fulfill this role, it can lead to a threat to male identity characterized by household conflict, resistance to sharing domestic work, control over women’s income, and violence [22,25]. The COVID-19 pandemic made both women and men more vulnerable by disrupting their work, increasing time at home, and raising care responsibilities. This situation led to more gender-based violence but also created opportunities for families to reconsider and sometimes change traditional gender roles [24,25,26]. Understanding how the pandemic affected informal female vendors requires a critical analysis of the associated societal transformations and the changes in household power dynamics. While crises often strengthen patriarchal norms, they can also create opportunities to renegotiate gender roles as families adapt out of necessity.

Despite growing literature on COVID-19’s impacts on businesses and urban food systems, there is less focus on how informal female-owned businesses in secondary cities, such as Kisumu, are affected. Kisumu’s strong links to rural communities and the thriving informal economy, mostly led by women, make it different from larger and primary cities with reference to its unique geographical and socioeconomic conditions. Although women, particularly those in the informal sector, continue to witness sociocultural injustices given the dominant cultural practices, and more so during emergencies, such as the pandemic, limited to no studies have focused on this minority population in the city. To address this knowledge gap, this study examined how the pandemic affected female informal food businesses in Kisumu. Specifically, the study answered the question “how do gender relations, the added caregiving roles, and experiences of violence shape both women’s crisis responses and recovery pathways during emergencies?” Using a community-based participatory approach with qualitative research approaches, the study highlights the ongoing sociocultural and structural barriers that limit the long-term recovery of informal food businesses. The evidence from this work will be useful in planning for any future pandemics and/or emergencies.

## 2. Materials and Methods

**Study Area**

This study was conducted in Kisumu City, Kenya. More details on the study site and methods are in our previous publication [24]. Kisumu is located on the shores of Lake Victoria and is Kenya’s third-largest city, serving as a vital commercial and transportation hub in the western region [33]. According to the 2019 Kenya Population and Housing Census, the city’s population was slightly over 600,000 people, with the metropolitan area, including Maseno and Ahero, totaling 1,155,574 residents [34]. Over the years, Kisumu has experienced population growth, resulting in an increase in informal settlements located near the city center. Despite its strategic location and resource base, Kisumu faces significant socio-economic challenges, with over 60% of the city’s population living in informal settlements. HIV prevalence is also relatively high in the city, affecting more than 15% of the population compared to the national prevalence of 3.3% [35].

Informal trade, agriculture, and fishing activities are the primary economic drivers in Kisumu city. In recent years, the city has experienced significant growth in informal settlements that house a diverse population, ranging from low-income earners to the unemployed, along with a few middle-income residents [20,30]. These areas often lack sufficient infrastructure and services, leading to challenges in sanitation, water access, and housing. The city also has a significant population of female informal food vendors, whose businesses serve as primary sources of income and contribute to local food security. Over 75% of the food consumed in the city is obtained from informal female traders [36]. Yet, regulations and post-emergency recovery strategies remain blind to the needs of women vendors in the city. We undertook this study to explore effective strategies to support recovery, especially in the aftermath of challenges like the COVID-19 pandemic.

**Study participants**

Participants in this study included informal food vendors from five major markets in the city: Manyatta, Obunga, Nyawita, Bandani, and Nyalenda and key informants from the local organizations and city government employees who work closely with informal vendors. We recruited a total of 80 participants who engaged in focus group discussions [FGDs] (*n* = 40), in-depth interviews [IDIs] (*n* = 20, and key informant interviews [KIIs] (*n* = 20). About 16% (*n* = 13) of these were male, with 9 participants being male key informants and 4 male informal food vendors. Male participants were included in the study to add valuable perspectives on household and community life. Their stories revealed how they viewed their economic roles, their partners’ challenges, and shifting gender responsibilities during the pandemic. These insights deepened the understanding of how gender dynamics shaped recovery in the informal food sector.

**Study Design**

This study employed a community-based participatory approach [37], collaborating with Pamoja Community-Based Organization, which has worked in the Kisumu region for over a decade. A CBPR approach was adopted to allow for community engagement and participation in the research process from the design to the dissemination and implementation of findings. We used qualitative methods—IDIs, KIIs, and FGDs- to gather data on the post-pandemic recovery experiences and challenges faced by urban informal female food vendors in Kisumu City, Kenya. This approach was suitable for this study because it allowed for a comprehensive exploration of the participants’ perspectives, experiences, and coping strategies within their socio-economic context [38].

**Sampling techniques**

We employed purposive and snowball sampling methods to select study participants [39]. These sampling strategies were adopted to allow for the recruitment of individuals with comprehensive and pertinent information regarding the study. We focused on women and men food vendors representing the informal sector, and key informants who work closely with the vendors, specifically targeting those who operated as food traders/vendors or supported the vendors during and after the COVID-19 pandemic. Furthermore, key informants were chosen based on their roles in the community, their positions within the government, and their involvement with informal food enterprises.

**Data collection techniques**

Trained qualitative research assistants fluent in local languages collected data for this study using various qualitative techniques.

**Focus Group Discussions (FGDs)**

We conducted four FGDs, each consisting of 10 female food vendor participants, totaling *n* = 40 participants. Participants were recruited across the 5 markets (Manyatta, Obunga, Nyawita, Bandani, and Nyalenda), with each market having at least 10 participants in the FGDs. Participants were included if they operated a food business during the pandemic, lived in Kisumu during the pandemic, and were willing to give informed consent to engage in a group discussion. The FGDs lasted approximately 2–3 h and were audio-recorded with participant consent. The FGDs were facilitated by trained and experienced moderators familiar with the local context. Our FGD guide explored group-level themes such as shared recovery strategies, peer support networks, and perceptions of external assistance. This allowed participants to share and discuss their experiences collectively, revealing common challenges and group-level dynamics such as shared recovery strategies and peer support networks. We also examined female vendors’ experiences during the pandemic and the community support strategies they adopted. This method provided insights into how food vendors collectively responded to and coped with disruptions in their community during the pandemic.

**Key Informant Interviews (KIIs)**

We conducted *n* = 20 KIIs with representative government officials, non-government organizations, community leaders, and representatives of informal food vendors in the respective informal markets in the city. With the KIIs, we aimed to better understand the pandemic’s impacts on business, the health and well-being of people in the informal food sector, and their household food security status. The KII examined policies addressing challenges and opportunities for post-pandemic recovery. Additionally, they investigated the broader policy landscape, support mechanisms, and systemic issues affecting informal sector vendors’ recovery. We considered these perspectives essential for understanding systemic barriers and opportunities in supporting informal vendors’ resilience.

**In-depth Interviews (IDIs)**

We also completed *n* = 20 IDIs, consisting of 16 women and 4 men, to gain a deeper understanding of the personal impact of COVID-19 and subsequent recovery strategies. These individuals were also recruited from across the 5 markets in Kisumu city. We captured detailed accounts of experiences and the complexity of individual resilience and adaptability. See the previous publication for more details on the study methods [24].

**Data Analysis**

The interviews conducted in Luo and Swahili were first translated into English by the other (SO) and (DO), fluent in the local languages and English. The research team (authors SO, PO, DO, and EO) held multiple team meetings to carry out a thematic analysis of the data [39]. Following Braun & Clarke’s step-by-step approach [40], we identified themes and patterns and interpreted the themes within the qualitative data [41,42]. First, we reviewed the transcripts multiple times to create an initial coding framework. Next, we performed open coding to derive preliminary codes, which were later organized into overarching themes representing key participant experiences. The analysis involved an iterative process, refining codes and themes through discussion and review to maintain consistency and credibility.

Next, we utilized Atlas.ti V 24 software, Berlin, Germany to effectively manage and organize the coded data. We selected representative quotes to highlight the main themes and offer deeper insights into the experiences of informal food vendors during and after the COVID-19 pandemic.

To guarantee rigor and reliability in our analysis, we used triangulation to compare data from all three collection methods [43,44], IDIs, KIIs, and FGDs. Additionally, we had a peer debriefing (with authors JO & JK) that enabled our team to discuss emerging themes, challenge assumptions, and refine interpretations through constructive feedback [44]. This approach ensured consistency and enhanced the validity and credibility of our findings.

**Ethical Considerations**

Ethical approval was obtained from the University of Alberta Research Ethics Board (REB# Pro00138854) and Amref Health Africa’s Ethics and Science Review Committee (ESRC) (REF: AMREF ESRC P1670-2024) in Kenya. Written informed consent was obtained from all participants before interviews to ensure they understood the study’s purpose, their rights, and their voluntary participation. Confidentiality was maintained by assigning unique identification numbers, unlinkable to personal information. Interviews occurred in confidential locations chosen by participants. All participant information has been securely stored in an encrypted, password-protected University of Alberta Google Folder for the project’s duration and five years afterward.

## 3. Results

### 3.1. Socio-Demographic Characteristics of Study Participants

The study included 80 participants, comprising 83.8% women and 16.2% men. Participants ranged in age from 25 to 65 years, with a mean age of 40.3 years (SD = 8.5). Over half (52.5%) of the participants had below primary or primary-level education, with at least one in four (25.3%) possessing tertiary-level education. Most participants (61.2%) were married, had at least one child, and lived with extended family members, indicating additional care responsibilities during the pandemic. For the majority of the participants, their main source of income was informal trading, highlighting their dependence on informal food vending for livelihood and household needs sustenance (Table 1).

### 3.2. Thematic Analysis of Data

Our qualitative data analysis revealed several transformations occurring in informal trading businesses. These transformations create opportunities to cope with emergencies while generating new challenges tied to gender and societal expectations. Such challenges limit women’s ability to continue contributing to urban household food security and livelihoods. Table 2 summarizes the themes and sub-themes we developed from our analysis of participants’ narratives.

#### 3.2.1. Market and Household Transformations

*Changes in market dynamics and consumer behaviors*

The pandemic impacted the livelihoods and operations of female food vendors in various ways. The city government implemented measures in the markets, including closing major markets, which limited food vendors’ access and hindered their usual business operations. Participants noted a decline in sales attributed to measures implemented by city, county, and national governments. Particularly, regulations such as curfews, lockdowns, and restrictions on movement both within and outside the city affected supply and demand systems. Participants reported changes in sales as well as the supply of commodities.

*“Selling was a problem, you could go to the market and yet there were no buyers to make purchases, it was like a loss, you ended up consuming the commodities for domestic use, … The next day if you wanted to do the same business, you could not since you lack the capital and you already made no profits.”*

(FGD1-P3)

*Changes in household food access and security*

Many participants reported increased food insecurity in their households, forcing their families to depend on one meal a day with a reduced quantity, which was insufficient for all household members. With the school closures, children and other family members were at home full-time during the lockdown, as opposed to when children were attending school.

*“I have five children who are eating and schooling. So, when my children were just at home… we were really suffering and were only able to eat once a day. Sometimes we could have supper but no breakfast and lunch and vice versa. …There was a time we even wished to go back to the village.”*

(IDI-02)

*Pandemic-induced displacement*

Many participants reported incurring debts they could not repay for basic necessities for their families since their businesses were not profitable. Many women struggled to pay their rent, forcing some to relocate to rural areas or more affordable homes. Additionally, household income dropped sharply, leaving women with the financial burden as most of their partners lost their jobs during the pandemic.

*“I was selling chapati and beans next to the roadside. COVID 19 affected me, until there was a time I was not able to pay for my house rent, we could not get food to eat, my children could not go to school so they were forced to stay at home. When my children were at home, I was forced to move from where I was staying to a different house. I used to pay rent of 3000 shillings and moved to a different house where I’m paying 2000 shillings….”*

(IDI-02)

#### 3.2.2. Gender-Specific Transformations

*Changing caregiving and breadwinner responsibilities*

The pandemic highlighted the unequal gender responsibilities, with women shouldering additional burdens amidst the changing regulations. Most women continued to juggle household chores, caregiving, and their food businesses. Furthermore, women faced financial strain as many reported that their partners lost their jobs, leaving them to take full responsibility for all household needs, including those of their partners.

*“That one, I can say that women, actually women are overburdened. Sometimes I even wonder how they got that extra time to do the business. Because, okay, a woman wakes up at 5:00 A.M., they prepare the children for schooling. Then once they have done that, there are other house chores, which they also do, like washing the dishes…. milking, milking for those who are in the village, milking the cows, fetching water, fetching firewood, preparing meals again, lunch for the children.…and the husband. By then, the husband is at the trading center playing [ajua] Luo board game, or gossiping.”*

(KII-10)

In addition, some of the men reflected on their own economic vulnerabilities, feelings of inadequacy linked to job loss, and the social pressures of maintaining their role as breadwinners as expected by society.

*“Livelihood was not good as business was low during the pandemic. I had to reduce what I provided at home so that I did not weigh down the business. As long as the man provides, he supports the wife’s business too. But when the business is low, everything is affected. You feel the pressure because the family still depends on you.”*

(IDI-18-Male)

These experiences show how the pandemic changed traditional gender roles and expectations within households. These insights suggest that recovery efforts should focus not only on empowering women but also on reshaping men’s roles and attitudes that contribute to gender inequality in the informal sector.

*Heightened gender-based violence during COVID-19*

The pandemic intensified existing patterns of gender-based violence, with many participants reporting increased conflict and emotional or physical abuse as economic hardship and household stress deepened. The financial pressure in the households also cultivated an environment for gender-based violence (GBV). Some participants reported that they had frustration that extended to their family members, including their children and even parents.

*“Lack of money in the house led to marital feuds. I know women who quit marriages because their husbands’ vented frustrations on them”*

(KII-04)

*“There were arguments as money is what causes peace, without money, there is no peace, this caused my separation from my husband, and at that particular time, I had a daughter who was to sit for her form four (Grade 12) exams but could not go to school as I could not afford to pay her fees…. We had an altercation with the father so he could not help, the child has never gone back to school to date. There were a lot of arguments, and my daughter ended up running away and so was my husband”*

(FGD 3-P5)

*Reinforced male patriarchal behaviors*

The pandemic reinforced these traditional expectations, as male participants described struggling to maintain their provider roles despite financial hardship, while many resisted sharing caregiving or household responsibilities. On the other hand, male participants provided a contrasting perspective, emphasizing the pressure of being the sole breadwinners. Some men reported facing immense stress as they struggled to provide for their families during a period of reduced income. For instance, a male participant reported that he had to find another alternative construction job after losing his primary source of income during the pandemic to sustain his family. Additionally, male food vendors highlighted the challenges they faced in sustaining their businesses amidst the pandemic and rising costs of supplies. One respondent noted the difficulty of balancing business expenses with household needs, often sacrificing personal meals to ensure their families were fed.

*“Yes, I would give money but not enough sometimes. And it depended on how I made my daily sales. So, she would be forced to just buy the food and see how to serve the children and then share the rest of food. We eat in the same plate and the food would just be enough for all of us.”*

(IDI-17)

Men also discussed household responsibilities, with some indicating a traditional division of labor where their spouses managed domestic chores. However, a few men mentioned stepping in occasionally to support when their wives were unwell or overwhelmed. One respondent expressed a willingness to take on more domestic tasks in the future if needed, recognizing the importance of shared responsibilities during difficult times.

*“When she is sick, I can look for someone to help her out because, with my work, I get injuries on my hands, though such responsibilities should be shared. So, if I get someone to help her that also helps.”*

(IDI-16)

Other males resisted shifts in gender roles and were unwilling to engage in household activities culturally or societally assigned to women. This resistance from male partners to participate in household activities underscores the cultural perceptions of masculinity and femininity, as shared by one of the female participants in the FGDs:

*“…It was tough because when you tell your husband to keep the fire burning in the (githeri) he would be like because you are feeding me now, I have to do those odd jobs that women are the ones supposed to be doing. If it is that food just do not give it to me just eat with your kids. But those womanly things I cannot do. So, it was hard.*

(FGD-01)

The pandemic highlighted gender inequalities, illustrating how men struggled with their roles as primary providers and the need to diversify income sources. For women, it underscored the importance of support systems to alleviate the combined pressures of caregiving and earning a living.

#### 3.2.3. Overcoming COVID-19 Related Transformations for Survival

*Survival through self-resilience*

Participants reported various coping mechanisms they used to ensure their businesses continued running despite the challenges faced during the pandemic. Most participants changed their business operations. For instance, some altered their business hours while others shifted to selling fewer or non-perishable goods, such as dried cereals.

*“So, it forced us to wake up at 4:00 A.M. so that we could afford to get our daily bread. … So I had to look for money, food, and how kids would go to school.”*

(FGD 1-P5)

Other coping mechanisms included operating the business from home or moving from door to door, especially for those selling cooked food. This helped them reduce their rent payments for a shop and avoid paying taxes. Some participants reported paying bribes to the police during curfew hours to secure permission to continue their business operations beyond the designated curfew period.

*“…people changed tact in how they did business. Some changed their locations from Kibuye market to within their homes while some even closed down”*

(IDI-15)

*“Police had it easy. Police followed me to the house, and since it was a curfew, I had to pay a fine of one thousand shillings. …”*

(FGD 3-P5)

To keep the business active and running, women utilized informal credit networks from fellow women or relatives. They also relied on customer trust to give out their goods on credit, which they would later repay.

*“Sometimes your regular customers would come to you without any money, and they would want to take the goods on credit because people had challenges during corona. … when my regular customers come to me to buy goods on credit, I would not refuse, instead, I would give them.:*

(IDI-17)

On the other hand, men also emphasized strategic financial planning within their households. Many worked closely with their spouses to prioritize essential expenses like food and school fees. This collaborative approach was crucial in ensuring survival during the pandemic.

*“We could sit down and agree, if I got some money, we could pay school fees, and pay electricity and water bills”*

(IDI-16)

*Survival through community resilience and support*

Community structures, including local market leaders and Community Health Volunteers (CHVs), played a crucial role in navigating the crisis. For instance, market leaders established rules during the pandemic to maintain hygiene and safety in their work areas. The CHVs provided general health education to food vendors on how to take precautions during the pandemic. Most participants appreciated this practice, as they gained knowledge to identify some signs and symptoms of COVID and how to handle cases.

*“During that period, we used to visit them in their houses to assess whether they considered and practiced the health messages that we taught them. We equally assessed to ensure that no sick trader went about their businesses in the market, wearing masks, and the distribution of masks that were provided by the government. In the distribution of masks, we prioritized the traders because the interacted with a multitude of people.”*

(KII-07)

Community-based organizations such as Shining Hope for Communities (SHOFCO) offered support during the pandemic to informal traders and urban households. The organization supplied water in tanks at the market and provided soap to food vendors, enabling them to comply with COVID-19 regulations and receive some financial aid to boost their businesses.

*“SHOFCO was really handy, I am a member, and we received KShs. 3000 ~ ($USD23) monthly on top of soap, flour, rice, maize, cooking oil and beans.”*

(IDI-14)

During the pandemic, food vendors formed smaller groups to facilitate security arrangements during uncertain times. They achieved this through collective contributions from each vendor willing to participate. However, despite the support they received from the community, participants noted gaps in institutional support, with many expressing frustrations over limited government assistance despite promises of aid.

#### 3.2.4. Post-Pandemic Recovery Pathways and Challenges

All participants noted that recovery has been gradual but uneven among different food vendors. Participants reported difficulties in regaining customers and adapting to inflationary pressures. Rising transport costs were also mentioned by participants as contributing factors to a slower recovery process. Political instability and increasing inflation further complicated the recovery for the food vendors. Participants noted that all these challenges stemmed from the pandemic. To sustain long-term recovery, they advocated for reduced fuel costs, which directly influence supply prices.

*“To ease the burden of those who are doing business, the price of fuel should be reduced. If the fuel price is reduced, all the prices of goods will also be reduced.”*

(IDI-17)

*“… Let us talk of demonstrations/protests. When protests are there, you see now we have to close the business and that particular day or the days you are making no income.”*

(KII 10)

To avoid similar vulnerabilities, some participants recommended enhancing local agricultural investment to lessen reliance on external suppliers, thereby ensuring resilience against future disruptions.

*“It is said that there is a lot of wealth in the soil. We need to invest in farming the returns can be bumpy and can make a difference in our lives.”*

(IDI-14)

Other participants suggested that the government should allocate emergency loans for food vendors to help cover economic shocks to businesses during unforeseen crises like the pandemic.

*“I think the emergency loans could help because at times someone has only a little cash and would be praying for anything to boost her so that would be great.”*

(FGD1-P4)

## 4. Discussion

This study set out to explore the impacts of the pandemic with reference to gender relations, the added caregiving responsibilities, and the experiences of violence on the informal female food vendors, their response to the crisis, and the adopted pathways to post-pandemic recovery. This was community-based research that adopted a qualitative research approach to allow vendors to share their experiences with the pandemic, the different changes and transformations in their daily activities due to the COVID-19 pandemic. The findings from this study reveal how women experienced different forms of transformations, beginning with the market and household adjustments, gender-specific transformations, and transformations in the coping strategies. At the market and household level, the female vendors experienced financial hardship with additional caregiving responsibilities during the pandemic. There was also increased food insecurity in the household during the pandemic, as most of the informal vendors could not operate their businesses to generate income and supply fresh and healthy foods to urban households. Families rationed and/or skipped meals to cope with the limited income and food supply. This is consistent with the findings from different studies across primary cities such as Nairobi and Accra, which noted increased hunger in informal settlements in urban households [18,22,23]. Despite the challenges that these food vendors went through, they showed remarkable resilience. Many food vendors opted for various strategies that worked for them in order to sustain their business. This included closing the business to reopen after the pandemic. Many food vendors adapted their business models by venturing into non-perishable goods and relocating their business operations to their homes to cut down on the rent payments. These findings mirror those from Wertheim-Heck and colleagues [27] who mentioned some of the adaptability among urban vendors facing disruptions. Others shifted to door-to-door sales and relied on informal credit loans to sustain their business, as indicated by Kimani-Murage and peers [45].

Furthermore, the findings highlight the diverse impact of the COVID-19 pandemic on gender roles and economic dynamics in the informal economy. The pandemic not only deepened pre-existing inequalities, including GBV, but also provided a unique lens to examine and challenge traditional gender norms. Our findings show that the pandemic intensified existing gender inequalities within the informal economy. Women faced increased responsibilities, balancing household chores, caregiving, and business operations. In addition, reports of GBV and distress highlighted the compounded vulnerabilities women face in times of emergency due to the prevailing sociocultural systems. Gender-specific challenges emerged, with women taking on increased responsibilities while continuing to support their households financially. This double burden, as highlighted by Crush and Tawodzera [46], exacerbated the strain on the physical and emotional well-being of the women. In addition, these findings also align with [19], who emphasized the disproportionate impact of crises on women’s mental health and economic stability. This highlights the structural challenges women face, as they are often expected to assume additional roles during crises without adequate support systems.

While the study centered on women’s experiences, male participants offered important views on changing gender roles during and after the pandemic. Many men spoke about their financial struggles, feelings of inadequacy after losing jobs, and pressure to remain breadwinners as defined by society. Their stories show how the pandemic disrupted traditional household roles and expectations, leading to different transformations within and outside the home. In some instances, the pandemic-related changes led to positive changes, including an increased interest in sharing care and household responsibilities. This suggests that recovery efforts should focus not only on women’s empowerment but also on redefining masculinities that reinforce gender inequality in the informal sector and in households. Future research should explore these dynamics further.

For men, the increased economic strain revealed their vulnerabilities as primary providers. Most males faced significant income instability and were compelled to explore alternative income sources, demonstrating the critical importance of diversifying livelihood strategies to withstand economic shocks. These findings align with broader literature on the disproportionate economic impact of crises on male breadwinners in patriarchal societies, where societal expectations intensify the pressures of providing for households [47]. The pandemic gave a picture of the need to re-evaluate and reallocate household responsibilities. Some men were willing to help with domestic chores during stressful times, but traditional norms often prevented lasting changes in gender roles. This points to the need for targeted interventions, such as awareness campaigns or community programs, to promote gender equity in both economic and household responsibilities. These gendered experiences underline the need for targeted interventions to address the specific needs of women and men in the informal economy. In addition, the pandemic highlighted systemic gender inequalities and created an opportunity to push for policy changes. However, such opportunities have not been utilized to address the prevailing inequalities in communities and at the government level.

From the findings, community support and collaboration were identified as crucial in surviving the crisis. Community health volunteers (CHVs) delivered health education, making the food vendors comply with the safety measures introduced by the Ministry of Health. This helped to reduce the spread of COVID-19. In addition, market leaders enforced hygiene regulations in the business operation areas, and some organizations, such as SHOFCO (Shining Hope for Communities), provided essential support, such as the provision of water tanks and soap in the markets. Food vendors mentioned forming informal groups, which fostered collective security and resource sharing, demonstrating the importance of grassroots collaboration in mitigating the impacts of the COVID-19 crisis. Vendors expressed that working together enhanced their ability to withstand economic pressures and navigate uncertainties. These findings align with the study done by Pimentel and colleagues [8], who emphasized the importance of collaborative initiatives in enhancing food systems’ resilience. However, as echoed by [48], gaps in institutional support, especially from the government, left many food vendors feeling unsupported. Despite widespread acknowledgment of the informal economy’s importance, especially in food security, vendors were excluded from financial aid, capacity-building programs, and infrastructure investments. Most of the informal vendors rely on individual and collective agency through social connections and support to cope with emergencies such as the pandemic.

To strengthen adaptations for future crises and economic shocks, a collaborative stakeholder approach is key. While grassroots collaboration proved effective, integrating these efforts with institutional support can enhance sustainability and equity. Our study does not provide a comprehensive policy analysis, but the lived experiences of vendors and local officials shared through this study underscore the urgency for more inclusive, practical, and accessible support mechanisms. Policymakers should prioritize creating formal mechanisms that link informal vendors to social safety nets, affordable credit, and capacity-building opportunities. Expanding the role of community-based organizations can effectively address local needs and strengthen future crisis response and recovery. Policy and decision makers should adopt a collaborative approach by bringing various relevant partners together to enhance resilience, strengthen social safety nets, and provide training on financial management to vendors.

### Study Limitations

This study is not without limitations, and the findings should be interpreted with caution. The study employed a cross-sectional design that restricts the exploration of the long-term recovery pathways and, hence, the need to conduct longitudinal studies. The study’s geographical focus on Kisumu City may limit the transferability to other cities. Although Kisumu City has its own unique social, economic, and political context, the qualitative findings offer valuable insights into the experiences of informal food vendors during and after COVID-19 that could be applicable, if not transferable, to other secondary cities with similar characteristics. The qualitative evidence in this study provided an in-depth contextual understanding of the lived experiences of informal food vendors, which was the focus of this study. Future mixed methods research that includes quantitative data is recommended to strengthen the findings by quantifying the impacts across the larger population. Additionally, our data collection was based on participants’ self-reported recollections, which may be subject to recall bias, inaccuracies, and social desirability influences. To minimize these limitations, we triangulated data across different population segments and used multiple data points—interviews, FGDs, and existing literature. We also conducted peer debriefings within the research team to validate emerging themes and interpretations. Despite these measures to enhance rigor, we acknowledge that some reporting bias may remain and are important considerations in interpreting the findings.

## 5. Conclusions

The COVID-19 pandemic exposed the vulnerabilities of female informal food vendors in Kisumu County while also highlighting their resilience and recovery strategies. The socioeconomic impacts experienced by these vendors underscore the critical need for targeted policies to support informal economies during a crisis. Female food vendors not only help in sustaining their families, but they also contribute significantly to community food security and livelihoods, therefore demonstrating their role in urban food systems. The pandemic served as a wake-up call, highlighting systemic disparities while offering opportunities to re-evaluate gender roles, improve support systems, and build a more resilient informal economy. There is a need for policymakers, community organizations, and other stakeholders to collaborate to create sustainable recovery pathways, addressing both immediate needs and long-term structural issues to ensure the survival and growth of this critical sector.

## Figures and Tables

**Table 1:** Demographic characteristics of study participants.

Characteristics	All (*n* = 80) Frequency (%)	KIIs (*n* = 20) Frequency (%)	IDIs (*n* = 20) Frequency (%)	4 FGD (*n* = 40) Frequency (%)
Education				
Primary or less	42 (52.5)	2 (10)	14 (70)	26 (65)
Secondary	17 (21.2)	3 (15)	4 (20)	10 (25)
Tertiary	21 (26.3)	15 (75)	2 (10)	4 (10)
Marital status				
Single	10 (12.5)	0	5 (25)	5 (12.5)
Married	49 (61.2)	14 (70)	12 (60)	23 (57.5)
Divorced/separated/widowed	21 (26.3)	6 (30)	3 (15)	12 (30)
Age categories				
18–29 years	8 (10)	0	4 (20)	4 (10)
30–49 years	58 (72.5)	14 (70)	14 (70)	30 (75)
50+ years	14 (17.5)	6 (30)	2 (10)	6 (15)
Gender				
Female	67 (83.8)	11 (55)	16 (80)	40 (100)
Male	13 (16.2)	9 (45)	4 (20)	0

**Table 2:** Analytic themes.

Analytic Themes (Themes)	Sub-Themes	Manifestation of the Themes
Market and household transformation	Market dynamics & consumer behavior Household for access/security Pandemic-induced displacement	Changes in market dynamics and consumer behaviors—reduced demands & supply Changes in household food access and security Poverty-induced displacement
Gender-specific transformations	Increased caregiving burden New face of GBV Reinforced male patriarchal behavior	Changing caregiving and breadwinner responsibilities A new face of gender-based violence Marital conflicts due to financial pressure
Overcoming COVID-19 transformations	Individual agency/Self-reliance for survival Community resilience & support	Survival through self-resilience Survival through community-resilience and support
Post-pandemic recovery pathways	Unequal recovery among vendors Rising costs and inflation Contextual issues—politics and policy issues	Rising inflation and increased cost of transport limit recovery Political unrest/protest is affecting recovery

## Data Availability

Data supporting reported results in this study are hosted on the University of Alberta Google Drive and are not publicly available currently, but are available on request.
